# Analyzing the contributions of a government-commissioned research project: a case study

**DOI:** 10.1186/1478-4505-12-8

**Published:** 2014-02-05

**Authors:** Ingrid Hegger, Susan WJ Janssen, Jolanda FEM Keijsers, Albertine J Schuit, Hans AM van Oers

**Affiliations:** 1 National Institute for Public Health and the Environment (RIVM), P.O. Box 1, Bilthoven BA 3720, The Netherlands; 2 Department of Health Sciences and the EMGO Institute for Health and Care Research, VU University, De Boelelaan 1085, Amsterdam 1081 HV, The Netherlands; 3 Tranzo Scientific Center for Care and Welfare, Tilburg University, P.O. Box 90153, Tilburg, TL 5000, The Netherlands; 4 Netherlands’ Organisation for Applied Scientific research TNO, P.O. Box 2215, Leiden 2301 CE, The Netherlands

**Keywords:** Alignment effort, Commissioned research, Contributions, Contribution mapping, Horizontal alignment, Interaction, Organizational environment, Public health, Vertical alignment

## Abstract

**Background:**

It often remains unclear to investigators how their research contributes to the work of the commissioner. We initiated the ‘Risk Model’ case study to gain insight into how a Dutch National Institute for Public Health and the Environment (RIVM) project and its knowledge products contribute to the commissioner’s work, the commissioner being the Health Care Inspectorate. We aimed to identify the alignment efforts that influenced the research project contributions. Based on the literature, we expected interaction between investigators and key users to be the most determining factor for the contributions of a research project.

**Methods:**

In this qualitative case study, we analyzed the alignment efforts and contributions in the Risk Model project by means of document analysis and interviews according to the evaluation method *Contribution Mapping*. Furthermore, a map of the research process was drafted and a feedback session was organized. After the feedback session with stakeholders discussing the findings, we completed the case study report.

**Results:**

Both organizations had divergent views on the ownership of the research product and the relationship between RIVM and the Inspectorate, which resulted in different expectations. The RIVM considered the use of the risk models to be problematic, but the inspectors had a positive opinion about its contributions. Investigators, inspectors, and managers were not aware of these remarkably different perceptions. In this research project, we identified six relevant categories of both horizontal alignment efforts (between investigators and key users) as well as vertical alignment efforts (within own organization) that influenced the contributions to the Inspectorate’s work.

**Conclusions:**

Relevant alignment efforts influencing the contributions of the project became manifest at three levels: the first level directly relates to the project, the second to the organizational environment, and the third to the formal and historical relationship between the organizations. Both external and internal alignments influence the contributions of a research project. Based on the findings, we recommend that research institutes invest in a reflective attitude towards the social aspects of research projects at all levels of the organization and develop alignment strategies to enhance the contributions of research.

## Background

For knowledge institutes such as the Dutch National Institute for Public Health and the Environment (RIVM), it is important to know what factors influence the impact of their research. This article aims to give insight into the process of a government-commissioned RIVM research project and the relevant alignment efforts needed to enhance its contributions to the commissioner’s work.

The RIVM is an independent knowledge institute with expertise in the fields of public health, infectious diseases, healthcare, medicines, lifestyle, nutrition, and environmental safety. Being a governmental institution, the RIVM conducts research on behalf of other governmental organizations to support them in their policymaking and supervisory tasks. One of the RIVM’s principal contracting agencies is the Health Care Inspectorate (hereafter: Inspectorate), which supervises the quality of health services, prevention measures, and medical products in the Netherlands. In a yearly program cycle, the Inspectorate submits knowledge questions to be answered by research conducted by the RIVM (Additional file 1 Yearly cycle for RIVM research in commission of the Health Care Inspectorate). For commissions to the RIVM, the Minister of Health puts a dedicated budget at the Inspectorate’s disposal, which means that the Inspectorate cannot use this budget for other purposes. Although RIVM investigators and inspectors interact during all phases of the research project, the Inspectorate, as the commissioning body, does not have authority over the research methods used, nor the outcome of studies as is laid down in the Act on the RIVM [1].

These days, the Inspectorate has to account for the effectiveness of its supervisory methods [2]. In its long-range plans, the Inspectorate expresses its objective to develop and use scientific knowledge for evidence-based supervision [3,4]. Consequently, research projects commissioned to the RIVM are often intended to contribute to the scientific basis of the Inspectorate’s work.

An evidence-based approach assumes that use of knowledge will lead to better professional practices resulting in both better and more legitimate outcomes [5,6]. This assumption strengthens the investigator’s expectations with regard to how research products will be used: investigators will produce scientific knowledge for the professionals, who will be eager to use it to improve their professional practices. However, this expectation often does not come true: utilization of research findings is difficult to achieve in practice. By now, the widely recognized difficulties in actual contributions of research have led to extensive research on knowledge utilization in healthcare and public health. Knowledge institutes use the insights from this research field in specific strategies to enhance the likelihood that their research will indeed contribute to improvements in practice; we call these strategies alignment efforts [7]. The RIVM also recognized the importance of alignment efforts and established them into procedures and guidelines on an institutional level. Some examples of such official alignment efforts are periodic meetings between managers of the RIVM and the commissioning organizations, training of project leaders where they learn to keep in touch with the commissioner’s contact person, and guidelines for the periodic progress reports and final RIVM products.

The RIVM’s aim is to support the Inspectorate in its supervisory tasks and tries to enhance its contribution to the Inspectorate’s work by specific alignment efforts. However, it often remains unclear for RIVM investigators to what extent their research really contributes and how its contributions could be improved. As a part of an RIVM strategic research project on improving knowledge utilization, we initiated a case study to explore how a RIVM project proceeded [8].

The objective of this study was to gain insight into how an RIVM project, including its knowledge products, contributed to the Inspectorate’s work and to identify the alignment efforts in the research project that influenced the research process and its contributions. In this article, we first present our study framework, the method used, and our findings in a ‘map’ of the RIVM research project. We describe the research project’s contributions and the alignment efforts that influenced the project. We finally reflect on the method used, especially on the feedback session with inspectors, investigators, and managers, as well as on the study limitations. In our conclusions, we provide also suggestions for improving research projects and its contributions.

### Study framework

**Figure 1:**
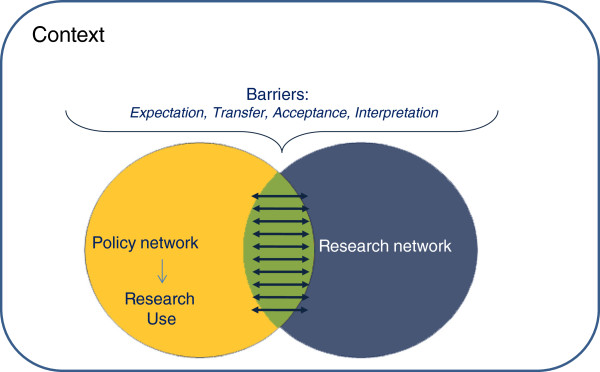
**The interaction model by de Goede et al. (2010) [**[9]**].** De Goede et al. visualized the distinct research and the policy networks as partially overlapping circles. In the Interaction Model, policymaking is an interactive and incremental process and extensive interaction between investigators and policymakers promotes research utilization. In the overlap of these networks, interaction takes place between the policy network and research network promoting research utilization.

The starting point for our study was the awareness that interaction between investigators and the intended users of research is generally considered the decisive factor to enhance the contribution of research. Based on the Interaction Model for research utilization in the field of health policymaking, de Goede et al. [9] developed an analytical framework that consists of a distinct policy network and a distinct research network. In the overlap of these networks, interaction takes place between the policy network and research network promoting research utilization (Figure 1).

In a way, this model also describes the context of RIVM research for the Inspectorate: both are distinct organizations with their own networks and they overlap at different levels. The two networks ‘RIVM’ and ‘Inspectorate’ specifically overlap in the research projects commissioned by the Inspectorate. Both organizations recognize the importance of interaction during the research project for the contributions of a research project as reflected in institutionalized alignment efforts.

Although the Interaction Model by the Goede et al. [9] is an adequate model to describe the overall relationship between the RIVM and Inspectorate organizations, this model did not offer a method to zoom in on the overlap of the two networks to analyze and explain the course of a research project. We needed a model for the complex research process with many different actors on different organizational levels evolving in a certain period. Contribution Mapping (CM), recently formulated by Kok and Schuit [7], offers a useful framework for our study, taking into account the social context of a research project and contains a three-phase process model of the research process. CM also includes a practical method for analysis, which we applied to our case study.

**Figure 2:**
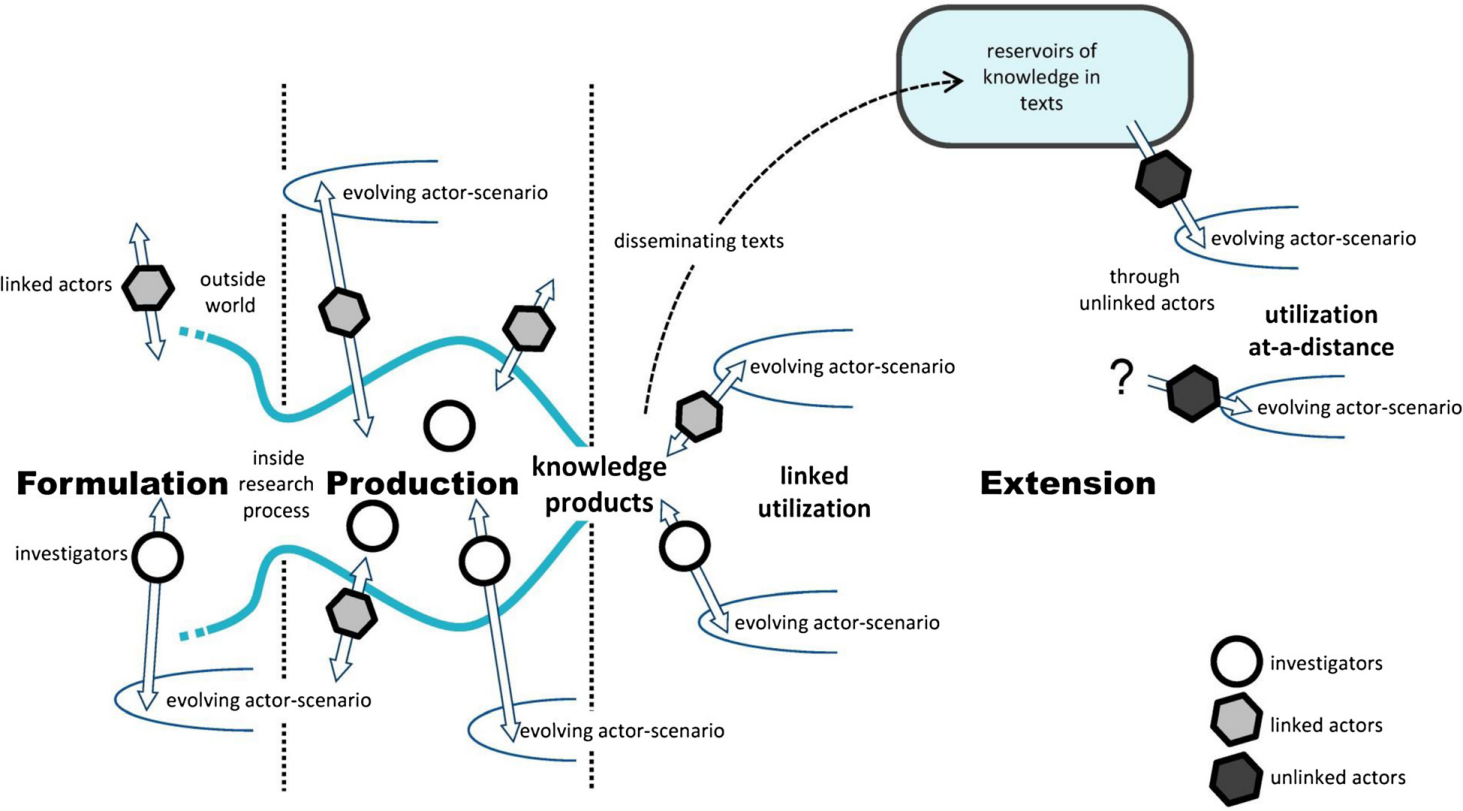
**The three-phase process model by Kok and Schuit (2012) [**[7]**].** We cite the description of Kok and Schuit to clarify the figure: “The three-phase process model. In the graphical representation of the three-phase process model, the two vertical lines separate the three phases. The search process narrows when a research proposal is formulated. At the beginning of the production phase, the search processes may widen again, before they are narrowed and the knowledge products are realized. During the production phase, there may already be some dissemination and uptake of emerging knowledge. After the knowledge products have been realized, the extension phase commences with dissemination and utilization in evolving actor-scenarios. Investigators are inside the research process, while linked actors are outside but able to interact in the process. Both investigators and linked actors can connect the research process to evolving actor-scenarios” [7].

During the research process, the research network and policy network form a hybrid world (Figure 2). Research is a process of co-creation where actors are, on the one hand, the investigators who perform the research and, on the other, the linked actors who in some way are connected to the project. Investigators and linked actors go through a joint process of co-creation in a research project. In this process of knowledge production, the three-phase process model distinguishes three demarcated phases in which typical activities take place:

i. Formulation phase: activities to initiate the research process, including funding, prioritization, and commissioning;

ii. Production phase: activities to realize the knowledge products;

iii. Knowledge extension phase: activities to make the knowledge available to potential users and to initiate and stimulate utilization.

It is difficult to assess the final impact of research on action for health and to measure the actual use of research products. A direct link from a research product to improved practices is often hard to trace. To handle this attribution problem, Kok and Schuit introduced the concept of contribution to action: “Contribution to action refers to the activities which turn novel combination of knowledge into a ‘going concern’, as part of practices, a component of successful innovation, as an element in decision making and their implementation” [7]. All phases of the research process can be relevant for contributions to health practices and Kok and Schuit distinguished four categories of research-related contributions: 

i. Changes in abilities and actions of involved and linked actors due to the research activities;

ii. The contributed knowledge products as such;

iii. Contributions through the utilization of the produced knowledge by investigators and linked actors;

iv. Contributions through utilization at distance by non-linked actors.

In research projects, actors are usually aware of problems in research transmission and utilization. They therefore design alignment efforts; during all phases of the research process, both investigators and linked actors undertake specific actions to enhance the desired contributions of the research process and the likelihood that the research indeed contributes and that the research products are used. Actors have their own actor-scenario which may have (decisive) influence on the process in all phases and thus on the contributions of the research process, on the knowledge product and/or utilization of the knowledge product. In practice, actors are not only involved in one research project, but are part of their own network and organizational environment and their actor-scenario continuously evolves. Kok and Schuit describe knowledge utilization as “incorporating knowledge into an actor-scenario as a means of contributing to its strength and scope” [7]. For incorporation into an actor-scenario, research knowledge has to be translated into a form and content that fits the actor-scenario. Alignment efforts will be most effective to enhance contributions of research knowledge when investigators and involved actors recognize and take account of the different actor-scenarios.

Based on the three-phase process model, Kok and Schuit developed a method to systematically evaluate the complex research process and the contributions of the research to action. According to this CM evaluation method, the investigators of a research project and the linked actors, such as potential key-users, are interviewed after an initial document analysis. Based on the findings, a map of the research process is drafted taking alignment efforts and an estimation of the contributions into account. In a feedback session, the draft map is presented to relevant stakeholders to validate the findings and to identify inconsistencies. Next, the contribution map is finalized and used for learning, improvement, and accountability purposes [7].

In our case study, we used the three-phase process model of CM as a study framework and we used the CM evaluation method to investigate the RIVM project ‘Risk Model’, commissioned by the Inspectorate, and its contributions. We specifically focused on the interaction between actors, especially the investigators and key users, using the theoretical concept of CM that alignment is the precondition for the contributions of a research project. Alignment efforts to create alignment are important to enhance its contributions; Kok and Schuit described various alignment efforts such as “engaging potential users in setting research priorities, formulating research and interpreting results, employing double*-*role actors in research and disseminating research results to potential key-users” [7]. In our case study, we inductively identified the alignment efforts that appeared to be relevant for the RIVM-Inspectorate relationship.

## Methods

To explore the contributions of a RIVM project to the work of the Inspectorate, we used a qualitative case study approach, including document analysis and semi-structured interviews [10]. This case study concerns the Risk Model project, a small project with a limited number of involved actors commissioned by the Inspectorate during three consecutive years.

### Contribution map

For the document analysis, we manually analyzed documents from the project archives within the RIVM and relevant public documents of the Inspectorate in order to reconstruct the course of events with respect to the project.

We interviewed actors from RIVM and the Inspectorate involved in the project (n = 10), namely the investigators (RIVM project leader and another RIVM investigator), the key users (four inspectors), and two RIVM managers and two managers from the Inspectorate as relevant informants. We used a topic list consisting of items regarding the research process, the actors and their organizational environment, the interaction between actors, and the contributions in the three phases of knowledge production, all based on the CM evaluation method. An additional file shows this in more detail (Additional file 2 Topic List interviews actors Case Study Risk Model). The RIVM principal investigator, who was also involved in the Risk Model project, conducted the semi-structured interviews face to face. Each interview lasted approximately one hour and was recorded and transcribed verbatim.

After importation of the data into Atlas-ti version 6.2.25, the principal investigator analyzed the data by coding [11]. We based the code list initially on the conceptual framework and completed it with inductive codes. The coding of the interviews started while data collection was ongoing and the topic list for the interviews was iteratively adapted in order to achieve data saturation. Three other investigators independently coded two interviews and we validated the coding in an open-coding session (agreement 72%; response 70%).

Based on the documentary analysis and interview data, we constructed a map of the research project according to the method CM by assessing the course and contributions of the project [7].

### Feedback session

Next, we organized a feedback session with two inspectors, a manager from the Inspectorate, the RIVM project leader, and a RIVM manager, to present and discuss our findings. All those present gave their feedback on the draft assessment and discussed actions for improving contributions and interaction. This discussion was recorded and transcribed verbatim. Based on the findings and the outcome of the feedback session, we drafted the final version of the contribution map and we inductively identified six categories of relevant alignment efforts.

## Results: the research process and its contributions

In this section, we present the course of the research process based on the data collected by documentary analysis and the interviews. We identified contributions in three categories: the utilization of the produced knowledge by inspectors, investigators and their organizations (contributions through linked utilization), the project’s knowledge products (contributed knowledge products), and changes in abilities and actions by inspectors, investigators and their organizations due to the research project (change in involved and linked actors). Contributions through utilization at-a-distance, the fourth contribution category distinguished by Kok and Schuit, did not emerge due to the confidential nature of the knowledge products [7]. In brackets, we name the identified contributions and the occasions where we observed the presence of alignment efforts.

### Background of the project

**Figure 3:**
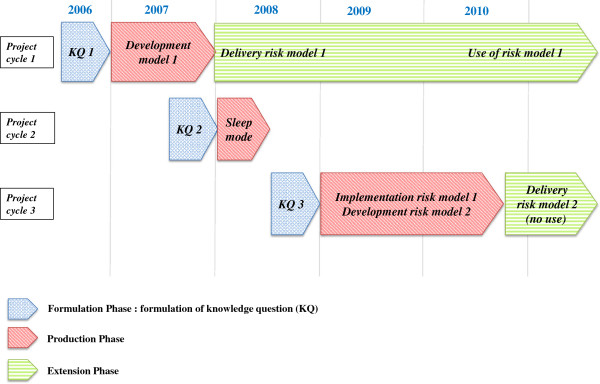
**Timeline for the risk model project.** Timelines of the Risk Model project visualizing the formulation, production, and extension phase for the three project cycles. In the formulation phases, the knowledge questions (KQ) were: i) the development of risk model 1 (KQ 1), ii) the implementation of risk model 1 (KQ 2; postponed to project cycle 3), and iii) the implementation of risk model 1 and the development of risk model 2 (KQ 3). In the production phases, the following activities took place: i) the development of risk model 1 (production phase 1), ii) no activities, project in sleep mode (production phase 2), and iii) the implementation of risk model 1 and the development of risk model 2 (production phase 3). Risk model 1 has been used in extension phase 1, risk model 2 has not been implemented or used.

The Risk Model project lasted for three consecutive years with an extension in the fourth year (2007–2010; Figure 3). According to the yearly cycle for RIVM research, the Inspectorate submitted a knowledge question for every project year to which the RIVM made a corresponding offer. For every project year, we distinguish the three phases according to CM:

i. The period for submitting the knowledge question and writing the project plan in an RIVM offer (from July until December; in the preceding year; formulation phase);

ii. The period for performing the planned research activities (from January until the delivery of all agreed knowledge products; production phase);

iii. The period after delivery of agreed knowledge products (extension phase).

The project aimed to develop a risk-based approach for clinical trial inspections in the Netherlands and had to deliver risk models to enable ranking and stratified selection of clinical trials for inspection. The research activities included the identification of risk factors and risk indicators and the development of two risk models: one for clinical trials on medicinal products (1^st^ year) and a second for clinical trials on food substances (3^rd^ year). We consider the models contributed knowledge products according to CM. To create these knowledge products, a synthesis of scientific knowledge on risk-ranking, risk models, medicinal products, and food substances was made and combined with legal and practical knowledge on clinical trials and inspections. This resulted into a new risk-based approach to clinical trials. For this project, the RIVM performed desk research in close collaboration with inspectors of the Inspectorate, without involvement of other organizations.

### Development of the first risk model

During the first formulation phase, the RIVM project leader and the contact person of the Inspectorate agreed to focus on clinical trials on medicinal products (alignment efforts; Formulation Phase 1).

Although in line with the official policy plan of the Inspectorate, the inspectors emphasized that it was not the driving force behind their research question; they felt the need for a tool for risk-based selection of inspection objects because of the overwhelming number of clinical trials they had to supervise (about 1,800 approved protocols each year). Although the inspectors were of the opinion that they had the expertise and competencies themselves, they had no time to develop a method for risk-based selection. They formulated an official RIVM knowledge question including distinct ideas for the approach of the problem and the design of a tool.

Inspectorate Manager 1: “*The knowledge question came up because of the enormous amount of inspection objects and the small number of inspectors involved. They tried to find a sensible way for risk-based selection.*”

At the RIVM, the origin of the research question was less clear. Some RIVM respondents thought the Inspectorate’s managers ordered the risk-based approach because of the official policy.

Researcher 1: “*I think that the knowledge question came from higher quarters, because of the general wish to have more risk-based supervision, just because of the general principle and to have a model for it.*”

After the formal start of the first production phase, it took several consultations to refine the research question and to agree upon the way to go (alignment efforts; Production Phase 1). This exploring phase in the production phase took longer than foreseen by the RIVM investigators in the project plan and caused delay. The data accessibility appeared to be much more problematic than anticipated, because the Inspectorate could not directly access the clinical trial database that is in control of another governmental organization, which largely hindered the development of the risk model.

The research team consisted of three RIVM investigators, who cooperated closely with three inspectors. During meetings, they discussed the scientific approach of risk ranking, risk factors, indicators, and draft versions of the risk model (alignment efforts). The project resulted in an electronic risk model and a report on its development (contributed knowledge product).

The transfer of the knowledge product by mail was without any project-specific dissemination activities and six months later than originally planned (Extension Phase 1). After transfer, the inspectors mentioned to the investigators that their management criticized the risk model. However, the inspectors internally solved the issue and the nature of the criticism never became clear to the investigators. In the interviews, the Inspectorate’s managers expressed some critical remarks on the scientific approach of the risk model but also mentioned that the inspectors involved were contented with the model and that this was decisive for accepting the knowledge product.

Researcher 1: “*And I became aware that manager 2 at the Inspectorate had a negative opinion and we asked for more explanation, but we never received it.*”

Since the risk model was considered an internal confidential inspection tool, the Inspectorate requested to keep the model confidential. The RIVM agreed although it meant that they could not further publish it nor use it in scientific discussions on risk-based supervision (alignment efforts).

On their side, the inspectors did not disseminate the risk model, neither in their external network due to its confidential status nor within the Inspectorate, because they were of the opinion that the risk model was too specific for other disciplines within the Inspectorate. The only occasion they exchanged the risk model was during the discussion with another governmental organization to get access to the clinical trial database (contributions through linked utilization). Nevertheless, they shared their experiences with the model in international discussions on risk-based inspections (contributions through linked utilization). The Inspectorate also mentioned the existence of the risk models in public presentations, in publications, such as annual reports, and in a formal answer to questions of the Parliament (contributions through linked utilization).

### Project in ‘sleep mode’

During the second formulation phase (that coincided with the first production phase), the Inspectorate formulated a new knowledge question: their intention was to incorporate the risk model in their operating procedures and they asked for RIVM assistance in the implementation (Formulation Phase 2).

After the official start of the second production phase, it eventually became clear that the inspectors could not realize the implementation of the risk model due to shortage of staff at the Inspectorate. Consequently, they did not need RIVM-assistance and the project went into ‘sleep mode’ (Production Phase 2). In fact, the yearly cycle was broken due to unforeseen circumstances and the Inspectorate officially postponed this knowledge question to the next (third) year.

### Implementation and a second risk model

For the third year, the knowledge question included both assistance at the implementation of the risk model and the development of a second risk model for another category of clinical trials (Formulation Phase 3). In their offer, the RIVM investigators did not yet define the category of clinical trials for this second model, but mentioned two options as an example. They stipulated that the choice for the new category should depend on the availability of appropriate data after an inventory of available data*.*

An RIVM investigator assisted at the implementation of the risk model for clinical trials on medicinal products and adapted the original model in order to facilitate data import into the model (alignment efforts; Production Phase 3). In June, a short report was presented to the Inspectorate on the progress, including the adapted electronic risk-ranking tool for clinical trials on medicinal products (contributed knowledge product)*.*

For the second risk model, the investigators and inspectors initially disagreed on the category of clinical trials. Due to a serious incident in a Dutch hospital in the previous year, the inspectors wanted clinical trials on food substances to be the focus of the second model. The investigators preferred clinical trials on medical devices based on the analogy to medicinal products, more available data and the match with their own expertise. However, the Inspectorate’s reality is that they also have to take into account the public and political circumstances when setting priorities in supervision and they felt the need to develop a risk model for a category that was less obvious for the researchers. This caused some tension in the project between investigators and inspectors, which was eventually resolved by discussion (alignment efforts).

The development of the new risk model for clinical trials on food substances needed extra unforeseen time and was not delivered according to the project plan but in the following (fourth) year (contributed knowledge product). During the finalization phase, only the RIVM project leader was involved and occasionally consulting other investigators. The model was again confidential and not published on the RIVM website. The second risk-model on clinical trials with food substances was never used (Extension Phase 3).

Interviewer: “*What is your opinion about the second model, did you use it?*”

Inspector 3: “*No, I cannot say anything about that because we did not use it until now.*”

Interviewer: “*Could you indicate what the reason is?*”

Inspector 3: “*Mainly because of competition with other tasks or other inspections. There were other issues with priority: international inspections and the thematic inspection project. And because the priority of the second category inspection objects decreased, the incident was dealt with and the interest in these inspection objects diminished.*”

### Sequel to the project

The investigators and inspectors had differing views on the desired sequel to the project which uncovered differing views on the ownership of the knowledge products. On one hand, the investigators regarded the risk models as their own knowledge products or at least co-creations with co-ownership of both parties. They expected that further development of the risk models would also evolve in co-creation by RIVM and Inspectorate and that they would spontaneously receive feedback on user experiences with the model.

Researcher 1: “*…I have the opinion that they should exchange this kind of information spontaneously.*”

The inspectors presented their risk ranking experiences in EU expert meetings, but did not provide feedback to the investigators; they were not aware that international discussions on a risk-based approach were of high interest to the investigators. They regarded the risk models as their own products and they choose not to involve the investigators in the evaluation and further validation of the models*.* The confidentiality of the products conflicted with the investigators’ interest to publish the reports and to use the findings in international discussions. Although both RIVM management and the investigators agreed with the required confidentiality, these differing expectations were discussed neither at the start nor at the end of the project causing unarticulated tension.

### Appraisal of contributions

All inspectors were rather satisfied with the contributions of the project to their work. In their opinion, they had used the risk models as much as possible and they expected further use, validation, and development of the models in the future (alignment efforts). However, the need for linking the risk models directly to the clinical trial database became gradually evident after transfer, but access to the database remained problematic. Without linkage to the database, the risk models turned out to be too laborious. Nevertheless, the inspectors were determined to move on with the models in coming years.

Inspector 2: “*Yes, I feel positive about it. Especially inspector 3 used it and in that sense I have the impression that the worksheets, the Excel sheets, function properly.*”

The Inspectorate’s managers were positive about the project afterwards, merely because the inspectors as users of the models were positive (alignment efforts). However, they showed some reserve since the risk models could only be in full use if connected to the clinical trial database. Given that this feature was not yet realized, the risk models had not taken away the managers’ concerns about the supervision of clinical trials. They still felt the need to have ways to predict specific, yet unknown risks in order to be able to prevent incidents and they doubted whether the current feature of the risk models was suitable for this purpose.

Inspectorate manager 2: “*We should think more about what we want to know and what we want to do with it at the start. We consequently do nothing with the report. Although this was not a bad example after all, since the inspectors felt contented about it. But it can be improved.*”

On the other hand, the RIVM investigators assessed the contributions of the project to the Inspectorate’s work as minor. After transfer, they were insufficiently acquainted with the implementation problems at the Inspectorate. They had expected that the risk models would be used on a daily basis within two or three years.

Researcher 1: “*I expected that we would establish a model able to scan all clinical trials within a maximum period of two, three years.*”

Interviewer: “*For all trials?*”

Researcher 1: “*For all trials, yes, but to achieve that more thinking, more communication was needed and it had to be done faster. But it did not work out that way…*”

At the site of the RIVM, the project contributed to the implementation of their strategy to extend the research area of the centers specialized in the quality control of medicinal products. The required expertise on risk ranking was on the fringe of the traditional expertise of the RIVM centers involved and the project offered the opportunity for developing new expertise (contribution ‘change in involved and linked actors’). However, this contribution was hindered by the confidentiality of the risk models.

### Consultative structure

In this study, we paid special attention to the interaction in all phases of the research project and we also found that all interviewed persons in this case study recognized the importance of their interaction to enhance the contributions of the research project. We asked them to indicate which factors could improve the process and contributions, and they all spontaneously indicated that the relationship and interaction like meetings and continuous consultations are crucial for success in a research project.

During the phase of formulation, the investigators and inspectors had only limited interaction. The formal procedure agreed between the Inspectorate and RIVM does not facilitate in-depth discussion on the knowledge question and the project plan due to the very formal deliberations at management level, short time periods, and strict deadlines within the procedure.

Inspectorate Manager 2: “*The more the question is vague, the more RIVM is not able to make a proper offer. If it is too unarticulated, the RIVM should not accept the question. What would the offer be based upon, which expertise and how many research hours? We should have more interaction.*”

During the production phases, the consultations went on in a good atmosphere according to both parties. In meetings, the inspectors and investigators openly discussed the risk factors and drafts of the risk model (alignment efforts).

Inspector 4: “*It was according to an established pattern. We discussed what we as inspectors thought about the results so far and then the RIVM got the work again to improve (the drafts).*”

The frequency of the consultations between RIVM and the Inspectorate depended much on proactive organization, availability of persons, and priorities at the Inspectorate (alignment efforts). The interaction between investigators and inspectors often suffered from time constraints and the inspectors and investigators experienced this in a different way.

The inspectors considered the frequency of interaction overall rather high and satisfactory, especially in the first year. The investigators emphasized that the lack of time of the inspectors to give feedback on drafts of the risk model resulted in delay. Although the inspectors regretted that their own time constraints delayed the evaluation and further development of the model, they accepted this fact of life.

Inspector 2: “*At the start, I think the intensity was very adequate, meetings on fixed time points. It is my feeling that there was need for it. The meeting dates were planned, but only used if needed. At a certain point in time, my role changed and I did not take part in everything anymore and therefore it is difficult for me to assess. It is my impression that it was less frequent for the extra model; however, this was the second project and things were clear already. So, although less frequent, it was sufficient in relation to the need.*”

Interviewer: “*What is your view on the interaction with the inspectors?*”

RIVM Manager 2: “*With great difficulty, at least between RIVM and Inspectorate and I have the impression that the inspectors get so many stuff and small issues on their desks that they have difficulty to pay attention to structured campaigns and they spent a lot of time in acting in a reactive way. I suppose…, this is my impression.*”

### Organizational environment

At the RIVM, the Risk Model project was a rather isolated project that lacked solid embedding in the research organization because the project was unusual and small in terms of people involved and research hours available. Colleague investigators were busy with the regular, large projects and not very interested because of the limited link with their research activities. The investigators had a few deliberations with their management to monitor the progress of the project at formalized moments (alignment efforts). The managers controlled the finances and timely output, but maintained their distance to the project and only commented on drafts of the final report (alignment efforts). At management level, meetings between the Inspectorate and the RIVM only focused on general issues and the Risk Model project was never discussed. The managers discussed neither the contributions of the project nor a way out for the confidential status of the knowledge products in order to facilitate publication of findings.

Investigator 2: “*We worked rather solitary. I experienced it as an isolated project, detached, isolated. It was not attached to other projects at all. It was not interwoven with the RIVM network.*”

An important finding was the major influence on the project of the established relationship between both RIVM and the Inspectorate. In the interviews, the inspectors, investigators, and managers expressed strong ideas of the other organization and we observed divergent views on the mutual relationship. At management level, the budget system was always an important issue since the Minister of Health does not allow the Inspectorate to use their dedicated RIVM-budget for commissioning research to other organizations. The Inspectorate considered the system therefore as a forced sourcing system without competitors for the RIVM and without sufficient possibilities to influence the RIVM research proposals. This view is in contrast with the perception of the RIVM managers and investigators, who considered their research for the Inspectorate as working on a mutual governmental task and experienced the Inspectorate’s view as unjustified distrust of their organization.

Inspectorate Manager 1: “*There exists a very negative view on the RIVM. Not with respect to their quality, but because we have this forced sourcing at the RIVM.*”

RIVM manager 1: “*The Inspectorate relies on RIVM results, but the forced sourcing reflects the relationship and this causes lack of trust.*”

On their side, the RIVM managers experienced that lack of time largely determined the quality of the Inspectorate’s commissioning role often resulting in unarticulated research questions. Because the RIVM accepted all research questions, this often led to unsatisfactory contributions of research projects.

RIVM Manager 1: “*So it was a knowledge question and actually, you were told to elaborate it per definition. So the only choice we had, was to choose the way to deal with the knowledge question, but not whether you should deal with it at all, that was no consideration at that time.*”

## Discussion

### Relevant alignment efforts

Analyzing the course of the Risk Model project and as indicated in the findings, we observed alignment efforts made by both RIVM and the Inspectorate; investigators, inspectors, and managers tried to create alignment within the bounds of their reach, both according to the institutional procedures at stated moments and according to the circumstances in the project. The importance of well-organized interaction between the networks RIVM and the Inspectorate is recognized on an institutional level; the RIVM and the Inspectorate have agreed upon transparent formal procedures to streamline the yearly commissioning process and to monitor the course of the agreed research projects. The Risk Model project was managed according to these formal rules and procedures. De Leeuw et al. [12] described a classification of 30 different theoretical frameworks for dealing with action on the nexus of research, policy, and practice. They established three groups of seven categories of theories, one of them the category Institutional Re-design. This category comprises theories that acknowledge the importance of interactions between actors from different institutions, which results in the explicit management of these interactions by maintaining institutional arrangements and establishing institutional rules. In our view, alignment efforts by the formal procedures match with strategies in the category Institutional Re-design. For both RIVM and the Inspectorate, alignment rules and guidelines were top-down established and explicitly encouraged project leaders and contact persons to interact with their counterpart at the other organization during formulation and production phase of a research project. Their management boards jointly discuss and revise regularly the agreed procedures to improve commissioning process. For the extension phase, the RIVM procedures provided guidance and rules for the presentation and lay-out, formal approval by the management and the transfer of contributed knowledge products.

Despite these institutionalized alignment efforts, we observed difficulties that could be attributed to lack of alignment at the level of the research project. Based on the course of the project and the contributions as described in the results section, we inductively identified six categories of alignment efforts that were relevant for the project.

#### Relevance: discussing the research results and their relevance for the commissioner and attuning the presentation of the knowledge products to the needs of the commissioner

Our findings show that the investigators and inspectors translated the need for alignment mainly in alignment efforts on ‘relevance’. The importance of alignment was in fact well recognized by the investigators and inspectors in the Risk Model project; they developed the two risk models as much as possible in close collaboration. During the production phase, alignment efforts primarily focused on discussing the concepts and drafts for the risk models and on the design of the electronic tool. From the perspective of both investigators and inspectors, alignment efforts on relevance were crucial for successfully accomplishing the research project. The investigators had to account for both scientific quality and customer satisfaction and the inspector’s interest was to get timely knowledge products that fitted to their needs, which required alignment on the level of the scientific aspects and the presentation of the results.

Together, they also had to deal with the tension that exists in commissioned RIVM research projects; the RIVM has an independent status established by law and in principle, the commissioner cannot determine research methods, influence the outcome of studies or prevent publication of results [1]. At first sight, the formal independent RIVM position conflicts with the need to align on ‘relevance’ issues. Here, the concept of backstage and frontstage work formulated by Goffman is helpful to overcome this contradiction [13]. Bekker et al. [14] described the RIVM as a boundary organization in the context of the Public Health Status and Forecasts (PHSF) reports and the biannual Dutch Health Care Performance reports. As a boundary organization, RIVM projects act as intermediary entities into which organizations delegate actors to contribute to the research and where frontstage and backstage work are combined in order to enhance the influence of the reports in informing public health policy. At the frontstage, organizations present themselves in their formal positions, whereas at the backstage, informal alignment paves the way for the organizations’ accountability at the frontstage. By analogy with the PHSF, the RIVM research projects for the Inspectorate could also be considered boundary areas where research for the benefit of the Inspectorate is performed and investigators, inspectors, and their managers collaborate. From this perspective, the formal agreed procedures represented the frontstage work to account for the organizations’ roles in the commissioning process. The meetings between the investigators and inspectors and their informal interaction at a personal level can be considered the backstage.

#### Consultative structure: agreeing on and acting on the consultative structure

For the Risk Model project, a limited consultative structure existed that mainly focused on the production phase. Van Egmond et al. [15] described the infrastructure developed by the RIVM for the above mentioned PHSF report, indicating that the backstage was well-organized, and presented it as an example for successful boundary work. However, in the Risk Model project, a backstage area at the start of the new project hardly existed and backstage work was limited to a few phone calls. Ample opportunity for backstage interaction, already in an early phase of the research project, will enhance the identification of possibly influencing factors and trust between actors and thus improve the process and its contributions. For the third project year, the opportunity existed to discuss the next year’s project plan during the regular meetings, but this opportunity was not intensively used. This shows that backstage work is not only a matter of available time, but also of culture and relationship between organizations. As argued by Wehrens et al. [16], a well-functioning boundary organization needs “internal room to discuss different perspectives, goals and expectations (and to find a balance that satisfies everyone involved) while the legitimacy of the activities, products and projects is not questioned in the broader organizations of the participants.” For the case of the Risk Model project, the internal room for backstage work was limited due to priority setting in both organizations and the relationship between RIVM and the Inspectorate that interfered with open discussion. The transition from the informal backstage to the formal frontstage was not clearly marked since the project continued for several years and, each year, new backstage work had to be done. Inspectors had to take both frontstage and backstage positions at the same time, causing confusion at the side of the investigators. In fact, the backstage was not very well developed and the organizations did not sufficiently recognize its importance in relation to the frontstage.

According to Wehrens et al. [16], the backstage and frontstage of organizations have different functions but are interrelated and cannot exist independently of one another. In most cases, the frontstage is predetermined by official arrangements that can even be emphasized by a ceremonial public presentation of the knowledge product to the commissioner. However, in the Risk Model project, the frontstage was marginalized because of the requested confidentiality of the risk models. Because of this, the Inspectorate did not have to account for using the risk models, which distorted the backstage space and made the investigators confused about the demarcation of backstage and frontstage.

#### Goal and timing: discussing and agreeing on the formulation of the knowledge question, its origin, the question behind the knowledge question, and the underlying need for the knowledge products

For three consecutive years, the Inspectorate formulated knowledge questions for the Risk Model project. The procedure for commissioning research to the RIVM was rather formal, with strict deadlines, and included several management levels for approval. The management regularly updated the rules with the intention to improve the research projects and their contributions. These formal procedures are important for legitimization of the research projects, but offered limited opportunity for alignment efforts at the investigator and key user level in the formulation phases. Although the formal alignment efforts with respect to the goal and timing of the project were in line with the established procedures, several interviewees mentioned that more informal interaction during this phase of a research process could improve its contributions. These findings are in line with the critical key factors identified by De Goede et al. [9] to influence the expectations of research users. Early extensive alignment efforts between investigators and key users on the formulation of the research question using previous experience and researchers’ knowledge, exploring the actual needs behind the question, and taking into account study limitations is important to manage the expectations on the contributions and achievable timelines at both sides.

#### Tasks and authority: explicit discussing of the relationship between actors and agreeing upon input and role of actors, responsibilities, sharing of information and knowledge, authority over the contributed knowledge products, and the follow-up of the research project

In the Risk Model project, the RIVM project proposals, as presented in the formal offers, were designed according to the formal guidelines and approved by both the RIVM management and the Inspectorate. However, the guidelines did not explicitly require consideration of time investment by the inspectors, the ownership of the knowledge products, the exchange of confidential information, and the follow-up of the project, whereas these issues caused friction later in the project. The alignment strategies based on the Institutional Re-design approach were not able to meet the need for mutual understanding between organizations at project level. Another category of theoretical frameworks described by De Leeuw et al. [12] is the Blurring the Boundaries model. In this model, the actors of organizations involved learn about each other’s world by obtaining access to it. In our study, a notable finding was that the RIVM and the Inspectorate afterwards differently assessed the contributions of the project, the interaction during the research process, and the ownership of the knowledge products. Investigators and inspectors had different actor scenarios, but were not aware of this. This illustrates the need for more alignment strategies based on the Blurring the Boundaries model. Understanding each other will generate more trust and confidence, better recognition of perceptions and more open discussions on sensitive issues. Every actor in a research process has his/her own scenario and perspectives for the future of the research project contributions. If investigators are aware of the changing circumstances in the commissioner’s organization and understand the role knowledge and research projects have in the commissioner’s scenario, they will be better able to promote the research contributions.

#### Vertical alignment: sharing information within the own organization and embedding of the research project within the own organization

In the literature, the importance of horizontal alignment between research and key users is exhaustively described; horizontal alignment efforts are considered the key for enhancing research contributions. We observed that also the vertical alignment within the own organizations of both investigators and inspectors was a very relevant factor for the research process and its contributions. Some difficulties in a project with respect to tasks and authority, relevance, and organizational environment cannot be tackled by investigators and linked actors at a project level and will often ask for a combination of horizontal as well as vertical alignment efforts at different levels in the organizations. The embedding of Risk Model in both RIVM and the Inspectorate was limited and the management was hardly involved during the production process, while their alignment efforts focused on general issues and not on project-specific issues. We argue that explicit vertical alignment efforts could bring project-specific issues to the attention of the management level at which horizontal alignment efforts could really enhance the solutions. The required confidentiality of the risk models serves as an example of a fundamental issue where vertical alignment efforts were lacking. The Inspectorate desired confidentiality because publication would also mean that they had to justify the selection criteria in the risk models to outside stakeholders and that they could no longer diverge from the selection method. On the other hand, the RIVM’s task and interest was to deliver scientifically legitimate knowledge products and, to achieve this, both the necessity of stakeholders’ involvement and publication of the risk models should have been considered. The inspectors were bound to their own organizations’ policy and the investigators met the requirements of the commissioner. Vertical alignment efforts on project-specific issues would have identified the different views of RIVM and the Inspectorate at the appropriate organizational level at an early stage and could have resulted in a more grounded RIVM position on the ownership issue and scientific approach.

#### Organizational environment: aligning with respect to relevant conditions outside the research project influencing the relationship between investigators and linked actors and/or the research project, such as changing priorities, incidents, media-events, and relationship with other organizations

In the formulation phase, a new research project is dealt with as a well-defined assignment that can be planned and controlled by the project manager. In reality, the research project and its actors are part of a complex organizational environment that continuously changes and influences the course and contributions of the project. In the Risk Model project, we identified several unexpected influencing factors from the Inspectorate’s organizational environment, such as data accessibility, time investment by the inspectors, and a serious incident that influenced the second risk model. At the RIVM site, implicit, not openly discussed organizational circumstances, such as the project’s embedding in the organization and the historical relationship between RIVM and the Inspectorate, appeared to be influencing. On the one hand, both investigators and inspectors did not exactly know and understand the underlying feelings and motives of the other organization and their alignment efforts focused mainly on the scientific issues. On the other, the project’s backstage did not sufficiently facilitate reflection on the project to develop feelers for influencing factors from the project’s organizational environment. Conscious alignment efforts on the organizational environment would require more trust and understanding as argued in Blurring the Boundaries models combined with a well-developed backstage to accommodate discussions.

### Reflection on contribution mapping (CM)

The evaluation method of CM aims to offer a practical and realistic approach to evaluate the contributions of a research project and to analyze the alignment efforts. In CM, both key users and investigators have an active role by being interviewed and participating in the feedback session. Due to these characteristics, this method can be considered a fourth generation evaluation method [17]. The criteria for assessment of contributions and alignment efforts are not fixed beforehand, but are based on issues that emerge from the interviews. In our view, this responsive evaluation was useful to unravel a research project such as Risk Model, the contributions of which remained, on the face of it, unclear. To elucidate our questions on research contributions, a qualitative approach taking into account the actors’ own issues and their social process was needed to find clues for improvement on both organizational and project level. By using CM, we indeed gained an illuminating insight in the course of the Risk Model project. The interviews delivered a rich data set that disclosed the contributions and alignment efforts. When presenting these findings to inspectors during the feedback meeting, investigators and managers recognized the analysis and findings, which adds to the validity of the analysis. The feedback meeting gave opportunity to discuss the differing views, to exchange experiences, and to formulate concerted actions to solve identified issues.

We would like to comment that for a small project such as Risk Model, the number of interviewees can remain limited to get a complete overview. For a major project, we expect that this would require a proportional number of interviews with a corresponding workload. Depending on the aim, CM users could consider focusing only on specific key users in order to conduct the CM with achievable effort. Another possibility for selective application of CM is to choose a project as a typical example in a specific context. In this case study, we recognized patterns and issues that are directly applicable to other RIVM research projects as well.

The concept of contributions was very helpful to identify the (added) value of the project for the Inspectorate’s work. The project’s attributed value manifested itself not as expected, but could be demonstrated by the concept of ‘contribution’. The CM method does not focus on assessment of the contribution category ‘utilization on distance’. In our case, this was not a drawback because of our focus on contributions to the commissioner’s work to improve RIVM projects.

Although the first aim of the feedback session was to add information to the collected data from interviews and document analysis, we experienced that such a feedback session is an alignment strategy in itself. We drafted an abridged report of the discussion on which the participants commented. The consensus report included several recommendations for improvement of the knowledge production process to be taken aboard for future projects and a proposal for a pilot to test the recommendations in a number of new projects. We sent the report to managers within both organizations, and we therefore consider the feedback session as a very useful three-layer part of the case study; the scientific data collection layer where participants were able to add information or to comment on findings; an alignment strategy in itself since the participants solved issues during the discussion; and a method to improve the research process.

By joining a systematic evaluation session, participants can learn how to improve the research process [18]. Although in our case all participants valued the feedback session in this respect, we experienced that the formulated actions for improvement did not automatically result in better research processes in actual RIVM projects since the agreed actions were subject to the same alignment problems as the project and turned out to be more complex than expected during the feedback session. We argue that a carefully planned and managed follow-up would be the fourth prerequisite of a successful feedback session.

### Study limitations

We have to mention some limitations of this study. The principal researcher of this study was also involved as an investigator in the Risk Model project. As an RIVM investigator, she is also involved in other projects for the Inspectorate at present. On one hand, she had the advantage of having inside knowledge about the research process and the context of the case. To overcome the disadvantage of being biased by own experiences, the coding of the interviews was validated by three other researchers not involved in the Risk Model project. The research team intensively discussed the analysis of the findings. Moreover, the findings were also discussed with the participants of the feedback session. Combining all feedback, we conclude that consensus on the analysis exists within the research team.

In this article, we describe the first case in a multiple case study on the contributions of government-commissioned research projects. For this multiple case study, the different cases are selected to cover a variety of characteristics that may influence the research process and its contributions. This first case was a relatively small research project within the specific context RIVM-Inspectorate. This questions to what extend the findings can be generalized to other research projects. Our findings were largely in line with our expectations based on the study framework. According to CM, inspectors, the RIVM project leader and managers, and the Inspectorate discussed the contribution map in a feedback session. They agreed on the findings and identified general issues that also emerge from other research projects. Moreover, we presented the final contribution map in several scientific meetings in the RIVM. The influence of limited interaction, organizational environment, and relationship with other organizations is widely recognized, also by investigators operating in other expertise fields and working with other commissioners. Although this case study describes a specific and small project, we consider the findings relevant for government-commissioned research projects. However, we will have more insight in the generalizability of this case after completion of all cases in our multiple case study.

## Conclusions and recommendations

By analyzing the course of the Risk Model project and its contributions, we wanted to establish which alignment efforts are important for the RIVM-Inspectorate context in order to find clues for improving the contributions of RIVM projects. We identified six categories of relevant alignment efforts that can be undertaken at three different levels: at the first level, alignment efforts between investigators and linked actors; at the second level, alignment efforts related to the organizational environment of the project appeared also to be important; at the third level, alignment efforts between organizations as part of their formal and historical relationship.

At the first level, investigators and linked actors can improve alignment by continuous reflection on actor scenarios, awareness of mutual expectations, and open discussions with commissioners at all levels in the organization. This will ask for substantial efforts from all actors involved and will take precious (research) time for interaction on an ongoing basis.

At the second level, more awareness of the importance of the organizational environment could help to formulate an adequate response to challenges for the research project for which vertical alignment efforts can be essential. Since the vertical alignment efforts turned out to be of influence, the focus in a research project should not only be on the external alignment efforts. Attention for the role of internal interaction, both at the level of the project as well as management level, and a good institutional embedding could improve the research process and thus its contributions.

At the third level, alignment efforts are more difficult to deal with; the historical relationship of the institutes determines their views on each other at all organizational levels and thus influences the interaction during the research process. This ‘soft side’ of the project can be hard to manage and therefore a collective approach at all organizational levels should be developed. It could be useful to openly discuss the historical relationship and its influences on the interaction within, and even between, organizations. We suggest that both research organizations and commissioning bodies should invest time and energy in regularly organized reflection sessions where both researchers and managers openly analyze and discuss the relationship with other organizations. This also implies that maintenance of this awareness will be important since collective views and memory also influence new actors. If all actors are continuously aware of the historical context and relational aspects of their research projects, they could anticipate on the implicit consequences and improve their alignment efforts. At the strategic organizational level, alignment efforts should focus on the organizational image to increase trust and legitimacy in order to overcome historically determined, yet invalid frames.

Based on the findings, we recommend that a research institute should encourage a reflective attitude towards the social aspects of research projects at all levels in the organization. The first step for improvement is to validate the importance of the process factors for a new project; the second step would be to calculate in the need for time for these aspects of the research process at the start of the project. This will facilitate the third step to anticipate explicitly on alignment efforts during the research process. With respect to the evaluation of a research project, we experienced that CM offers an alignment effort in itself, apart from being a useful instrument to evaluate a research project. The feedback session as part of CM yielded not only information about the research process and its contributions but also elicited awareness and preparedness for improving research processes at the side of the participants. CM can therefore be a useful instrument in reflections on contributions and research processes. Nevertheless, one should be aware that a feedback session needs carefully planned follow-up in order to improve the research process; the jointly formulated actions are in fact new projects themselves and need no less alignment efforts than other projects.

Organizations, investigators, and all other linked actors should be encouraged to experience the profits of social investments in research projects and should be rewarded for doing so. To achieve this, different strategies based on different models should be considered. Institutional rules incorporated in the organizations ‘quality system’, can facilitate project teams, but should be coupled with sufficient room for well-organized backstage work with involvement of actors familiar in both organizations. The challenge is to convince organizations and their staff to consider alignment efforts as a valuable dimension of research projects, instead of extra burden, because of its influence on project contributions and on trust in and legitimacy of the organization in a broader sense.

## Abbreviations

CM: Contribution mapping; Inspectorate: Health care inspectorate; PHSF: Public health status and forecasts; RIVM: Dutch national institute for public health and the environment.

## Competing interests

The authors declare that they have no competing interests.

## Authors’ contributions

IH designed the study framework and methods, conducted, coded and analyzed the interviews, and drafted a first version of the manuscript. SWJJ, JFEMK, AJS, and HAMvO commented on the study framework, methods, analysis, and manuscript, in joint sessions and individually. All authors read and approved the final manuscript.

## Supplementary Material

Additional file 1 **Yearly cycle for RIVM research in commission of the Health Care Inspectorate.** Textbox describing the yearly research cycle of the RIVM: the formulation of the knowledge question by the commissioner, the corresponding RIVM offer, the research phase, and finally, the delivery of the agreed knowledge product. Click here for file

Additional file 2 **Topic list interviews actors Case Study Risk Model.** The topic list for the interviews consists of items regarding the research process, the actors and their organizational environment, the interaction between actors and the contributions in the three phases of knowledge production. Click here for file
