# Supplementary material for: Analyzing the contributions of a government-commissioned research project: a case study
Source: Health Res Policy Syst. 2014 Feb 5;12:8. doi: 10.1186/1478-4505-12-8 (PMC3946030; doi:10.1186/1478-4505-12-8)
Supplement: Additional file 1 — Yearly cycle for RIVM research in commission of the Health Care Inspectorate. Textbox describing the yearly research cycle of the RIVM: the formulation of the knowledge question by the commissioner, the corresponding RIVM offer, the research phase, and finally, the delivery of the agreed knowledge product. [file 1478-4505-12-8-S1.docx]

Every year, the Minister of Health puts a dedicated budget at the Inspectorate’s disposal to assign for research to the RIVM.

In July, the yearly cycle starts: the Inspectorate formulates numerous research questions for the next calendar year. For each individual question, the RIVM appoints a project leader and the Inspectorate appoints a contact person. The project leader and contact person interact to clarify the research question and to agree upon the desired research products.

After the clarification talks, the RIVM project leader drafts an offer for the research project that includes a concise project plan and the budget required. The RIVM management integrates the total package of all draft offers into a single official RIVM offer and submits this to the Inspectorate in September. The Inspectorate prioritizes the proposed projects and may ask for amendments in order to fit the final package into the Inspectorate’s research budget. By the end of the calendar year, the Inspectorate will finally accept the revised RIVM offer package.

The agreed research projects usually start at the beginning of the new calendar year. During the project, the RIVM project leader and the contact person have to keep in touch to monitor the progress of the project. The transfer of the research product takes place at the agreed moment during the calendar year.

In July, the cycle for the next year starts again.

During the research process, the contact person represents the Inspectorate. Most contact persons are university-educated inspectors with some research experience of their own. They are often one of the intended users of the research product and can be involved in the research project as experts.
