# Supplementary material for: Analyzing the contributions of a government-commissioned research project: a case study
Source: Health Res Policy Syst. 2014 Feb 5;12:8. doi: 10.1186/1478-4505-12-8 (PMC3946030; doi:10.1186/1478-4505-12-8)
Supplement: Additional file 2 — Topic list interviews actors Case Study Risk Model. The topic list for the interviews consists of items regarding the research process, the actors and their organizational environment, the interaction between actors and the contributions in the three phases of knowledge production. [file 1478-4505-12-8-S2.docx]

## Additional File 2 - Topic list interviews actors Case Study Risk Model

| Respondent | Topics | |
| --- | --- | --- |
| Position | Job description | |
| Experience | Period; previous functions | |
| Background | Education; school | |
| Role | Tasks; accountability | |
| Expertise | Expertise; specific area of interest | |
| Motivation | Personal interests in research project | |
| Knowledge need | Strategies to gather information (personal, organizational) | |
|  | |  |
| Formulation phase | | |
| Process | Participation and role respondent in formulation phase  Proceedings of the commissioning process  Knowledge question: origin, quality, relevance | |
| Actors and their network | Actors involved in commissioning process: investigators, linked actors  Characteristics actors: position, experience, expertise | |
| Context: organizational environment | Influence of the context at the time of commissioning: existence and relevance of organizational, political and societal issues.  Project’s relation to Inspectorate’s policy  Project’s relation to RIVM policy | |
| Interaction | Communication during formulation phase: internal and with other organizations  Interaction during formulation phase: actors, structural, planned, formal/informal, frequency, ways (telephone, meetings, e-mail) | |
| Contributions | Importance of the research project (organization; actor’s position; actor’s interest)  Use of knowledge question and commissioning research project (symbolic, instrumental, conceptual) | |

| Production Phase | |
| --- | --- |
| Process | Participation and role respondent: with respect to research content and research process  Proceedings: progress, notable events, problems, results |
| Actors and their network | RIVM project team: number, characteristics, selection, role, expertise, experience, participation meetings  RIVM network: actors involved in project, departments involved, , management involved  Inspectorate’s project team: number, characteristics, selection, role, expertise, experience, participation meetings  Inspectorate’s network: actors involved in project, departments involved, management involved |
| Context: organizational environment | Accountability for research project within RIVM and Inspectorate Position of the project teams within RIVM and Inspectorate  Importance of the research project within RIVM and Inspectorate: attention received, priority  Relevant political developments during the research project  Relevant societal developments during the research project  Influence media on the research project |
| Interaction | Communication during production phase: communication means (telephone, meetings, e-mail, indirectly)  Meetings: frequency, actors, issues discussed, structure, proceedings, character (formal/informal) |
| Contributions | Knowledge transmission during production phase  Intermediary/ draft knowledge products: characteristics, number, objective  Use of knowledge during production process  Opportunities for improvement of research process |

|  | |
| --- | --- |
| **Extension Phase** | |
| Process | Transfer knowledge products from RIVM to Inspectorate: procedure, form, communication, timing, dissemination within and outside Inspectorate / RIVM |
| Actors and their network | Actors involved in transfer knowledge products and scientific presentation of knowledge produced  Recipients of the knowledge products,: position, expertise, number, department |
| Context: organizational environment | Inspectorate’s requirements for transfer of the knowlegde products (confidentiality versus publicity, timing, communication)  RIVM’s requirements for transfer of the knowlegde products: format, internal release procedures, communication, confidentiality versus publicity)  Timing: in time, relevance for Inspectorate  Relevant political/societal developments in extension phase: during or just after transfer knowledge products  Media attention: scale, newspaper/television/internet  Simultaneous transfer of other knowledge products: RIVM products, products of other organizations |
| Interaction | Presentation knowledge products: method, audience, timing, media use  Interaction RIVM-Inspectorate after transfer: consultations, meetings, follow-up, respondents opinion on quality interaction |
| Contributions | Familiarity with project and knowledge products: receipt, reading, implementation, use  Contributions: agenda setting, budget, operating procedures, use of the knowledge products (instrumental, conceptual, symbolic)  Barriers for using knowledge products: usability, timing, actuality (expectations), language, other reports, media (transfer), fits with own needs, quality (acceptance), relevance, institutional interests (interpretation)  Respondent’s opinion on knowledge produced: quality, reliability, applicability, degree of contentment  Respondent’s opinion on knowledge products: quality, presentation, language, reliability, independence, relevance of recommendations, usability, degree of contentment  Opportunities for improvement contributions |
